# A Genome-Wide Analysis of a Sudden Cardiac Death Cohort: Identifying Novel Target Variants in the Era of Molecular Autopsy

**DOI:** 10.3390/genes14061265

**Published:** 2023-06-15

**Authors:** Livia Beccacece, Paolo Abondio, Arianna Giorgetti, Carla Bini, Guido Pelletti, Donata Luiselli, Susi Pelotti

**Affiliations:** 1 Computational Genomics Lab, Department of Pharmacy and Biotechnology, University of Bologna, 40126 Bologna, Italy; livia.beccacece2@unibo.it; 2 aDNA Lab, Department of Cultural Heritage, University of Bologna, Ravenna Campus, 48121 Ravenna, Italy; donata.luiselli@unibo.it; 3 Unit of Legal Medicine, Department of Medical and Surgical Sciences, University of Bologna, 40126 Bologna, Italy; arianna.giorgetti@unibo.it (A.G.); carla.bini@unibo.it (C.B.); susi.pelotti@unibo.it (S.P.)

**Keywords:** forensic genomics, autopsy, unexpected death, sudden death, sudden cardiac death, coronary artery disease

## Abstract

Sudden cardiac death (SCD) is an unexpected natural death due to cardiac causes, usually happening within one hour of symptom manifestation or in individuals in good health up to 24 h before the event. Genomic screening has been increasingly applied as a useful approach to detecting the genetic variants that potentially contribute to SCD and helping the evaluation of SCD cases in the post-mortem setting. Our aim was to identify the genetic markers associated with SCD, which might enable its target screening and prevention. In this scope, a case–control analysis through the post-mortem genome-wide screening of 30 autopsy cases was performed. We identified a high number of novel genetic variants associated with SCD, of which 25 polymorphisms were consistent with a previous link to cardiovascular diseases. We ascertained that many genes have been already linked to cardiovascular system functioning and diseases and that the metabolisms most implicated in SCD are the lipid, cholesterol, arachidonic acid, and drug metabolisms, suggesting their roles as potential risk factors. Overall, the genetic variants pinpointed herein might be useful markers of SCD, but the novelty of these results requires further investigations.

## 1. Introduction

Sudden cardiac death (SCD) is an unexpected natural death due to cardiac causes, usually happening within one hour of symptom manifestation. It generally occurs in individuals without any prior pathological condition or in good health up to 24 h before the event [1,2]. The aetiologies of SCD are heterogeneous, involving numerous diseases, and differentiated between age groups. In young people, the most common causes of SCD are complications of cardiomyopathies (i.e., hypertrophic, dilated, or arrhythmogenic cardiomyopathies), channelopathies (i.e., long QT syndrome, short QT syndrome, Brugada syndrome, or catecholaminergic polymorphic ventricular tachycardia), and even cardiac malformations. In contrast, in older people, SCD is mainly due to coronary artery disease (CAD) and, to a lesser extent, cardiomyopathies, myocarditis, and valve diseases [3].

The identification of SCD causes through autopsy examinations is sometimes not trivial and inconclusive. In these cases of sudden unexplained deaths (SUD), genomic screening is a useful approach to detecting the genetic variants that potentially contribute to SCD [4]. Since the first post-mortem genetic testing, also known as a “molecular autopsy”, it has been possible to discover the genes implicated in sudden cardiac deaths over last two decades, which is also thanks to massive improvements in sequencing technologies using small amounts of DNA in a cost-effective manner [1,5]. Molecular autopsies mainly include the screening of the genes known to be involved in cardiac arrhythmias, such as *KCNQ1*, *KCNH2,* and *SCN5A*, which are associated with long QT syndrome, and *RYR2*, which is associated with major catecholaminergic polymorphic ventricular tachycardia (CPVT) [4,5,6]. On the other hand, genome-wide association studies, which test greater numbers of genes, have allowed for the discovery of the novel genetic loci associated with SCD [5,7,8,9,10,11]. It is of note that SCD might be the result of the combined effect of several genetic polymorphisms and not only from a unique mutated gene [12].

In this study, we performed a case–control analysis using post-mortem genome-wide screening with the purpose of identifying the genetic markers associated with sudden cardiac death, which might enable its target screening and prevention. The hypothesis is that the genetic variants associated with sudden cardiac death are located in the genes known to be involved in cardiovascular diseases, such as the genes mentioned above, and in the genes that are part of the metabolic pathways involved in cardiovascular function and homeostasis.

## 2. Materials and Methods

### 2.1. Study Population and Controls

Thirty autopsy cases were included in this study. The inclusion criteria were: (a) cases submitted to a complete medico-legal investigation, including a full autopsy, cardio pathological examination, and systematic toxicological analysis at the Department of Medical and Surgical Sciences, University of Bologna, between 2018 and 2021; (b) deaths classified as a SCD; and (c) a post-mortem interval defined by autopsy as <5 days. The SCD investigations and diagnoses were performed according the 2017 guidelines of the Association for European Cardiovascular Pathology [13] by a medico-legal examiner after a comprehensive evaluation of all the post-mortem findings, which are better detailed in Section 2.2. The autopsy findings were grouped into three categories, as follows:

1. Normal heart, when no macroscopic and microscopic alterations were found;
2. Atherosclerotic coronary artery disease (CAD), when an acute coronary occlusion or severe atherosclerotic plaque with coronary luminal stenosis of >75%, in the absence of other acute diseases, was found;
3. Other “highly probable” CoDs such as cardiomyopathies, myocarditis, congenital coronary artery anomalies, and channelopathies, etc.

The following data were collected from the included cases: demographics (age, gender, and ancestral origin), medical history, with a particular focus on cardiac diseases, neurologic diseases, or infectious diseases connected to drug use, e.g., HIV or hepatitis C, cause, and manner of death. For the genetic analyses, the samples were pseudo-anonymized by assigning laboratory coding, followed by progressive numbers.

As a control cohort for the genetic screening, we selected individuals from the Tuscan population (TSI, Italy) of the 1000 Genome Project [14] and the Bergamo population (BERG, Italy) of the Human Diversity Genome Project (HGDP) [15]. We randomly chose twenty individuals belonging to the TSI and all the available individuals of the BERG population, for a total of thirty samples, five of which were females. Whole-genome sequences mapped to the GRCh37 primary reference assembly were recovered from online repositories of the projects.

Descriptive statistics were provided for all the data. The sktest was used to assess the gaussian distribution of the numerical variables. Depending on this, a parametric or non-parametric analysis of variance was used to test the age difference between the groups with different causes of death. The chi-square test was used to explore the association between the categorical variables (ancestry, gender, activity before death, comorbidities, and toxicological results) and CoD. The statistic tests were performed with Stata 15.1 (StataCorp LLC, College Station, TX, USA) and considered to be significant with *p*-values < 0.05. The figures were realized with Prism (GraphPad Software, LLC, version 9.0.0).

### 2.2. Post-Mortem Examination

A full autopsy was performed according to a shader forensic methodology [16]. The cardio-pathological analysis was performed according to the 2017 guidelines of the Association for European Cardiovascular Pathology by a forensic pathologist and expert cardio-pathologist [13]. When no certain or highly probable causes of death were found, initial genetic testing for 38 genes implicated in cardiac arrhythmia was performed, following an internal protocol for SCD [17]. During the autopsy, samples of urine, bile, peripheral (femoral) blood, or, in the absence of peripheral blood, aortic or heart blood, and other biological matrices, when needed, were collected. The blood specimens were preserved with 2% sodium fluoride. All the specimens were stored at −20 °C immediately following their collection during the autopsy. A general toxicological screening and quantification for alcohol, illicit drugs, and medicinal drugs were performed. The analyses for alcohol were performed using gas chromatography coupled to a Flame Ionization Detector (Shimadzu QP 2010 Plus, Kyoto, Japan). The blood samples were screened for cocaine, cannabinoids, opiates, methadone, and amphetamine-like drugs (amphetamines/methamphetamines/MDMA/MDA) using an immunoassay (ILab 650, Werfen, Barcelona, Spain) [18]. The confirmation analyses for cannabinoids were performed with a Shimadzu GC-2010 Plus gas chromatograph interfaced with a QP 2010 Ultra mass spectrometer (Shimadzu, Kyoto, Japan) [19]. The confirmation analyses for other illicit drugs and screening/confirmations for 68 psychoactive medications (benzodiazepines, Z-drugs, antipsychotics, antidepressants, and medical opioids) were performed with an ACQUITY UPLC^®^ System (Waters Corporation, Milford, MA, USA) equipped with an Acquity UPLC^®^ HSS C18 column (2.1 × 150 mm, 1.8 μm; Waters), following a previously validated method [20].

### 2.3. Genotyping and Data Quality Control

After the DNA extraction from the blood samples of the sudden cardiac death group, the DNA was genotyped for ~720,000 genetic markers using the HumanOmniExpress BeadChip (Illumina, San Diego, CA, USA).

The quality control on the sequenced variants was performed using a combination of the PLINK version 1.9 software [21] and Linux-based command line. The following filtering steps were applied:

- Retention of autosomal markers only;
- Removal of duplicate variants;
- Retention of variants with a missingness rate lower than 5% (--geno 0.05);
- Retention of individuals with a missingness rate lower than 5% (--mind 0.05);
- Retention of variants with values of probability for Hardy–Weinberg equilibrium test below the threshold of α = 0.01/number of markers, considering the Bonferroni correction for multiple testing/(--hwe α);
- Removal of variants with a minor allele frequency (MAF) lower than 0.01 (--maf 0.01).

### 2.4. Bio-Geographical Ancestry

To infer the genetic ancestry of the sudden cardiac death group, we carried out geographical contextualization against a dataset of 737 Italian individuals [22] typed for 550,000 genetic markers. The individuals were collected in 20 locations across the Italian peninsula, as well as in Sicily and Sardinia, using the grandparents’ criterion (both parents and all four grandparents must have been born in the same location as the sampled individual) to ensure that the local ancestry had been preserved. A Principal Component analysis (PCA) was performed after merging the SCD cases with the Italian control individuals and applying an extra set of filtering options with the PLINK 1.9 software [21], as indicated in the following list:

- Removal of variants with a minor allele frequency (MAF) lower than 0.01 (--maf 0.01);
- Removal of variants in linkage disequilibrium (LD), by computing pairwise linkage disequilibrium among markers in a sliding window of 50 single nucleotide variants, with a step of 5 variants and LD threshold of 0.1 (--indep-pairwise 50 5 0.1);
- Retention of individuals with a values of identity-by-descent (IBD) coefficient lower than 0.125 (--genome).

The PCA was performed by converting the PLINK dataset using the *convertf* software, followed by a computation of the principal components using the *smartpca* tool contained in the *eigensoft* suite of programs for population genetics (version 6.0.1) [23,24].

### 2.5. Data Processing and Statistical Analysis

A chi-square analysis was performed to find out the likely associations of alleles and genotypes with sudden cardiac death. Above all, an χ^2^ test with both one and two degrees of freedom was carried out on allele frequencies of almost 46,000 polymorphisms to identify the genetic variants that differed between the cases and control groups, potentially contributing to SCD. The genotypes of the variants with higher chi-square values were then tested for association by using the χ^2^ test with two degrees of freedom and the Fisher exact test. The two statistics were performed in a comparison between the subgroups of cases, identified by autopsy examination, and the controls, even comparing the subgroups to each other (intra-autoptic group comparison). A pathway enrichment analysis was carried out to assess the pathways enriched with the genes identified by the statistical analyses. The enrichment analysis was implemented on the R statistical software version 4.2.2, which runs Bioconductor version 3.16, through the R package *enrichR* version 3.1. The gene sets were retrieved from the KEGG, Gene Ontology, and WikiPathways using the Enrichr tool [25] accessed via *enrichR*.

## 3. Results

### 3.1. Post-Mortem Data Collection

**Figure 1:**
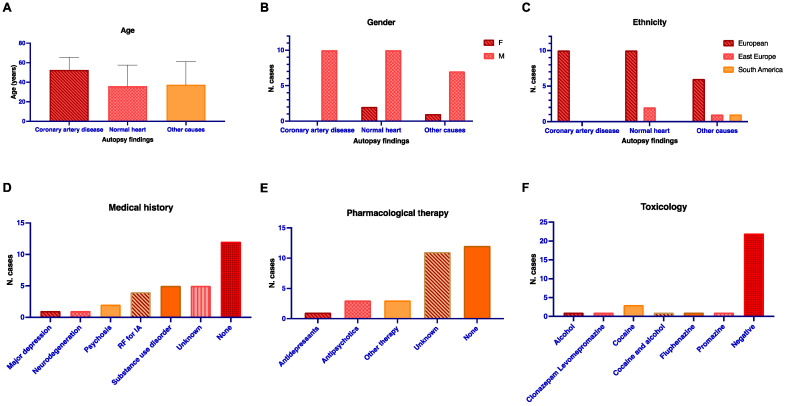
Age ((**A**), median and interquartile range), gender (**B**), and ancestry (**C**) as distributed according to autopsy findings groups are reported in the upper part of the figure. Number of cases divided according to medical history (**D**), therapy (**E**), and toxicology of the analyzed cases (**F**) are reported in the lower part. N: number. F: female. M: male. RF: risk factors for cardiovascular diseases. IA: ischemic attack. Other therapy: medications for diabetes and cardiovascular factors.

In total, 30 autopsy cases identified as SCD that underwent a full post-mortem examination at the University of Bologna were included in this study: 27 males (90%) and 3 females (10%). The age range was from 2 to 76 years old (mean 43.4, SD 19.9, and median 45.5). A total of 26 of the deceased (86.7%) were of European ancestry, 3 subjects were considered to be from the Near Eastern ancestry group (10%), and 1 subject (3.3%) was from South America. The past medical history included alcohol or drug use disorders in 5 cases (16.7%), psychiatric or neurological diseases in 4 cases (13.3%), and cardiovascular risk factors (obesity, hypertension, and diabetes) in 4 cases (13.3%). A negative history was observed in 12 cases (40%), and in 5 cases (16.7%), no clinical histories were available. Pharmacological therapy was present in 4 cases (13.3%) suffering from a neurological/psychiatric disease (1 with antidepressants and 3 with antipsychotics) and in 3 cases (10%) for the treatment of diabetes and/or hypertension. In 12 (40%) cases, no therapy was present, and in 11 cases (36.7%), these data were not available. The toxicological analyses of the blood detected the presence of psychopharmacological therapy in 3 cases (10%, fluphenazine, clonazepam, levomepromazine, and promazine). Alcohol was detected in 1 case, cocaine in 3 cases, and both alcohol and cocaine in 1 case. The toxicological analyses were negative in 22 cases (73.3%). In none of the positive cases were drugs found in toxic/lethal levels and SCD was considered the only cause of death, with a contributory role being identified, on the basis of the multidisciplinary post-mortem analysis, in 4 cases involving cocaine. As for the autopsy findings, a normal heart was found in 12 cases (40%); atherosclerotic coronary artery disease was found in 10 cases (33.3%); and other “highly probable” CoDs were identified in 8 cases (26.7%). The data and descriptive statistics results are summarized in Table 1. The categorical variables were not statistically associated with CoD (*p* > 0.05). Age did not show a normal distribution and did not differ within the groups of CoD, as demonstrated by the non-parametric analysis of variance. The median age, gender, and ancestry across the groups based on the autopsy findings, as well as the medical history, therapy, and toxicology of the cases, are shown in Figure 1.

### 3.2. Analysis of Data

The chi-square (χ^2^) test, performed on allele frequencies of ~46,000 SNPs (single-nucleotide polymorphism), identified more than 2000 variants with statistically significant differences in the frequencies (*p* ≤ 0.05) between the cases and controls, which might be associated with SCD. Among these variants, 356 SNPs, having the highest statistical values (*p* ≤ 0.001) between the analyzed populations, were selected for further investigation (Supplementary Table S1).

The top SNPs map inside or near 456 genes, both in coding and non-coding regions. The majority of these polymorphisms had not previously been implicated in any phenotype and disease; however, there were 25 variants that had shown a previous association with cardiovascular diseases and phenotypes that increase the risk of developing these pathologies (Table 2), confirming the hypothesis of this study.

Among the 356 top variants without any prior association to other phenotypes, some were located in genes already implicated in cardiovascular functions and diseases, such as *TBXAS1* encoding the thromboxane A2, which promotes vascular thrombosis [26]. Many of these genes are involved in the development of atherosclerosis and coronary artery diseases, which are risk factors for sudden cardiac death. We even found that some of these polymorphisms are in genes already associated with sudden cardiac death, namely *CACNA1C*, *KCND2, PRKAG2*, and *SREBF2* [9,27,28,29]; however, except for a variant in *SREBF2*, the polymorphisms in *CACNA1C*, *KCND2,* and *PRKAG2* did not show a previous association with this phenotype. All the genes implicated in cardiovascular diseases are reported in Supplementary Table S2.

In addition, by studying the functions of all the genes, we discovered that many of them are involved in brain functioning, neuropsychiatric disorders, and drug metabolism and dependence.

**Table 2:** Genetic variants showing previous associations with phenotypes increasing the risk of cardiovascular diseases.

Variant	Variant Type	Gene	Association
rs11220463	Intron variant	*ST3GAL4*	Total cholesterol and LDL levels, carotid intima-media thickness [30,31,32]
rs6693954	Intron variant	*REN*	Blood pressure in type 2 diabetes patients [33]
rs17222723	Missense variant	*ABCC2*	Doxorubicin-induced cardiotoxicity [34]
rs7905784	Missense variant	*MCM10*	Myocardial infarction risk [35]
rs3813867	2KB upstream variant	*CYP2E1*	Ischemic stroke, alcoholic liver cirrhosis [36,37]
rs9332119	Intron variant	*CYP2C9*	Warfarin dosage [38]
rs310831	Missense variant	*E2F7*	Venous thromboembolism [39]
rs1087	3′ UTR variant	*CPB2*	Fibrinolysis inhibition level [40]
rs938886	Missense variant	*TEP1*	Cardiac frequency increase in gastric cancer patients [41]
rs2985684	Missense variant	*DNAAF2*	Carotid intima-media thickness [42]
rs4775041	Intergenic variant	*LIPC*	Levels of triglycerides, HDL and total cholesterol [31,43]
rs1126464	Missense variant	*DPEP1*	Hypertension, homocysteine levels [44,45]
rs12986742	Intron variant	*LINC01122*	HDL levels [46]
rs2061347	Intergenic variant	-	Serum linoleic acid concentration in metabolic syndrome [47]
rs2228314	Missense variant	*SREBF2*	Hypercholesterolemia, atherosclerosis, sudden cardiac death [29,48,49,50]
rs3738000	Missense variant	*NEK11*	Carotid intima-media thickness [42]
rs1053239	3′ UTR variant	*CIDEC*	Hypertension, response to antihypertensive drugs [51]
rs1870377	Missense variant	*KDR*	Atherosclerosis, ischemic stroke [52]
rs9991328	Intron variant	*FAM13A*	Triglycerides and HDL levels [31]
rs619203	Missense variant	*ROS1*	Atherothrombotic ischemic stroke [53]
rs4148821	Intron variant	*ABCB4*	Alanine aminotransferase levels [54]
rs42524	Missense variant	*COL1A2*	Risk of sporadic intracranial aneurysm [55]
rs6472155	Intron variant	*CYP7B1*	Coronary artery disease risk [56]
rs4149264	Intron variant	*ABCA1*	Influence on statins effectiveness [57]
rs7853989	Missense variant	*ABO*	Risk of venous thrombosis, reduced clearance of coagulation factor VIII [58,59]

### 3.3. Pathway Enrichment Analysis

**Figure 2:**
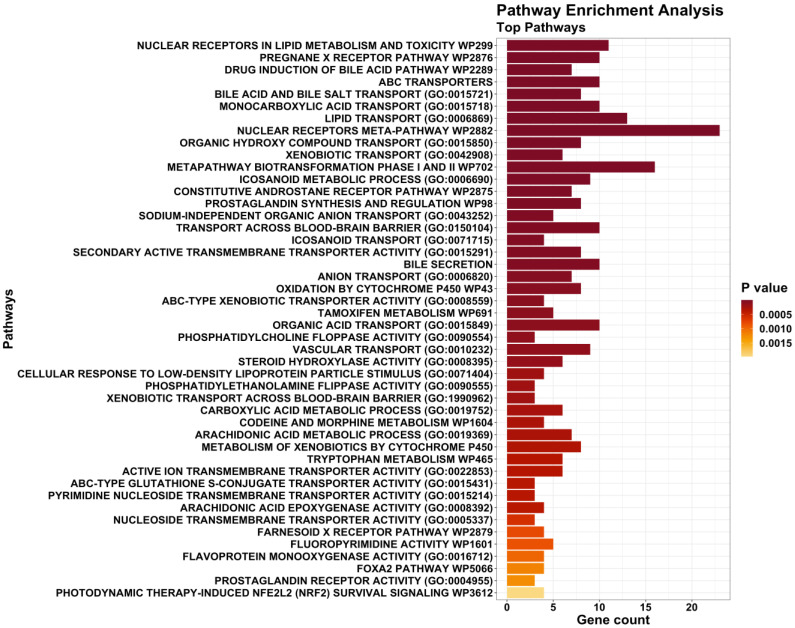
Most significant pathways enriched by the genes discovered through the chi-square test in case–control comparisons. All pathways displayed in this figure have the *p*-adjusted ≤ 0.05.

In order to identify the metabolisms most involved in and likely associated with SCD, a pathway enrichment analysis was carried out including 456 genes, where the 356 top variants were mapped inside or near. The analysis, performed via the R package *enrichR*, retrieved from KEGG, Gene Ontology, and WikiPathways 3548 pathways enriched by these genes, of which many were equivalent, owing to the different identifiers used by the three databases. Among these, 46 pathways, having *p*-adjusted lower than 0.05, were selected as the top pathways implicated in SCD (Figure 2, Supplementary Table S3). These top pathways are mainly involved in the lipid, cholesterol/bile, xenobiotics/drugs, and arachidonic acid metabolisms, which are known to be involved in cardiovascular disease development [60,61,62].

### 3.4. Association with Autopsy Findings

The forensic autopsy identified three subgroups based on the autopsy findings, which were coronary artery disease, other known CoDs, and normal heart. We therefore decided to verify if the three subgroups of sudden cardiac death cases differed in the genotypes of their top variants. The χ^2^ test with two degrees of freedom and Fisher tests were implemented on the genotype frequencies by carrying out a comparison between the controls and three autoptic subgroups, comparing the subgroups to each other. Overall, the tests identified the genotypes of 38 genetic variants with statistically significant differences in their frequencies (*p*-value ≤ 0.05), of which, 33 variants were in the subgroup–control comparison and 21 variants were in intra-autoptic group comparison (Table 3). The “other known CoD” subgroup had the greatest number of variants with statistically significant genotypes (32), followed by the “coronary artery disease” subgroup (5) and finally by the “normal heart” subgroup, with only 2 variants. In contrast to the other subgroups, the “normal heart” subset displayed genotypes with statistical significance only in the comparison with the controls. The autoptic subgroups differed from each other (Table 3), except for the rs6746883 variant, which showed significant statistical values in both the “coronary artery disease” and “other known CoD” subgroups with respect to the control group (Table 3).

Only the intron variant rs12986742 in the *LINC01122* gene, which was significant only in the “other known CoD” group, displayed a previous association with HDL levels (Table 2 and Table 3) [46].

## 4. Discussion

### 4.1. Case Control Study

Sudden cardiac death (SCD) is one of the leading causes of mortality in the world, and in Western countries, it amounts to nearly 20% of deaths [63]. The complex task of establishing the exact cause of SCD belongs to pathologists and many SCDs present a clear pathological cause, which can be detected and identified with varying degrees of confidence through a complete post-mortem examination. However, a high percentage of cases remain with an unexplained cause of death, despite careful macroscopic, microscopic, and additional toxicological and molecular analyses [1]. Post-mortem genetic testing, focused on cardiac-disease-associated genes, offers the opportunity to help in investigating cases of unexplained SCD and might improve the identification of the factors associated with arrhythmogenic risks or subtle structural abnormalities, even before the manifestation of pathological structural abnormalities [13]. The inclusion of a higher number of genes through genome-wide analyses allows for the detection of novel genes and variants, expanding the knowledge on SCD and providing biomarkers that are useful for prevention [5,7,8,9,10,11].

The genome-wide screening performed in this study through a case–control analysis allowed us to pinpoint many genetic variants with statistically significant differences in their frequencies (*p* ≤ 0.001), potentially contributing to pathogenesis of SCD, most of which showed no previous link with other phenotypes or diseases. Among these variants, 25 SNPs could be considered to be likely pathogenic for SCD in our study, as they were consistent with previous publications showing an association with cardiovascular diseases or other risk factors for the development of these pathologies (Table 2). In particular, the missense variant rs2228314 (Gly595Ala substitution) in the *SREBF2* gene, encoding a transcription factor that regulates the expressions of the genes involved in cholesterol biosynthesis [64], has been associated with the pathogenesis of coronary atherosclerosis and an increased risk of SCD, especially in middle-aged males [29]. Importantly, the cases analyzed here displayed a high frequency of the minor allele C (MAF = 0.7), which is the risk allele for SCD [29]. Atherosclerosis is a very impactful cardiovascular disease with a high mortality rate, characterized by chronic vascular inflammation as well as cholesterol accumulation, which highly contributes to its pathogenesis [65]. Atherosclerosis, in turn, leads to CAD [66], which is one of the main causes of SCD [67], and our study allowed for the confirmation of several variants related to CAD, but also to thrombosis and risk factors for atherosclerosis (cholesterol, HDL and LDL levels, intima-media thickness, and hypertension [65]) as useful markers of SCD.

Using a case–control study, many other polymorphisms were also pinpointed as related to SCD, mapping inside or near 456 genes with different functions. Although the majority of the polymorphisms identified herein have not formerly been associated with any phenotype, many of these SNPs were mapped in genes already implicated, to some degree, in cardiovascular system functioning and diseases (mostly atherosclerosis, CAD, and thrombosis, see Supplementary Table S2), indicating a likely relationship with SCD. Moreover, this link with SCD was strengthened by the presence of three genes (*CACNA1C*, *KCND2*, and *PRKAG2*) already associated with sudden cardiac death [9,27,28], as well as *SREBF2*. However, it is necessary to further explore the roles of these variants in the pathogenesis of SCD, especially considering the lack of previous associations with this phenotype. Furthermore, by deepening the roles of these genes, it was found that the most statistically significant biological pathways (*p*-adjusted ≤ 0.05) involved in SCD are represented by the lipid, cholesterol, arachidonic acid, and xenobiotics/drugs metabolisms (Figure 2). Overall, these results would further confirm our initial hypothesis, since these metabolisms have already been related to an increased risk of developing cardiovascular diseases [60,61,62], which may finally result in SCD.

The lipid, cholesterol, and arachidonic acid metabolisms are widely related to cardiovascular diseases. As mentioned, impaired blood levels of lipids and cholesterol are widely known to be risk factors for atherosclerotic plaque formation, the pathogenesis of CAD, myocardial ischemia, and ischemic stroke [61]. Arachidonic acid is a ω-6 polyunsaturated fatty acid that is metabolized in a class of bioactive molecules called eicosanoids (i.e., prostanoids, leukotrienes, epoxyeicosatrienoic, and hydroxyeicosatetraenoic acids), which are implicated in cardiovascular homeostasis, inflammation fostering, and even thrombosis [26,60]. The enhanced cleavage of arachidonic acid from cellular membranes triggered by pro-inflammatory stimuli and the consequent increased synthesis of eicosanoids, especially of prostanoids, have been associated with atherosclerosis, CAD, myocardial infarction, and thrombosis [26,60]. Among the genes involved in this metabolism, we detected three variants (rs6948035, rs17161326, and rs6962291) in the *TBXAS1* gene to be statistically significant in our population (*p* < 0.001). The thromboxane A2 (TxA2) encoded by *TBXAS1* plays a detrimental role in the cardiovascular system because it induces platelet aggregation, vascular dysfunction, vasoconstriction, and even cardiac arrhythmias [26]. Regarding the variants in *TBXAS1*, the intron variant rs6962291 has been related to aspirin intolerance in asthmatic patients and the minor allele A (MAF = 0.6333 in our study population) seems to reduce the degradation of TxA2 [68], suggesting that it could be a promising biomarker of sudden cardiac death. Overall, this evidence seems to confirm the link between thrombosis and SCD, as displayed by previous data [69].

Some of the cases examined herein (eight in total) were positive for medical or recreational drugs and it is noteworthy that we detected drug metabolism as one of most significant metabolic processes implicated in SCD, since there is evidence that many drugs can induce and exacerbate cardiac arrhythmias [62], which are a common cause of SCD, especially in young people [3]. The detected medical drugs (levomepromazine and clozapine) only displayed a weak or moderate association with QT prolongation [70] and no variant was detected in the genes that have a modulatory effect on membrane potentials, allowing us to exclude a synergistic effect of drugs resulting in sudden death.

In the case–control comparison, we identified many genetic variants mapping in the genes involved in drug metabolism, such as the intron variant rs1202171 in the *ABCB1* gene, which seems to influence the expression of other ABC transporters [71], and the missense variant rs17222723 in the *ABCC2* gene, which is related to drug-induced cardiotoxicity [34]. Both genes encode the transporters of the ABC family of transporters, which are involved in drug transport and highly associated with drug resistance [72]. It will therefore be interesting to deepen our understanding of the roles of the variants localized in drug-metabolizing genes in SCD and if they play a primary causal role, given that other polymorphisms in *ABCB1* increase the risk of sudden cardiac death in digoxin users [73]. Unfortunately, the case–control study performed herein did not allow us to analyze the effect of the variants within each single case, and thus to further explore the association of such variants with a subset of SCD due to possible antipsychotic/illicit drugs. Further studies on a wider casuistry might allow the investigation of this issue. Furthermore, a possible cooperation between the drug and arachidonic metabolisms in the pathogenesis of SCD has been highlighted, since some genes (for instance *CYP2E1*, *CYP2C9*, and *CYP2J2*) displaying variants with a statistical significance are involved in both metabolic processes (Supplementary Table S3).

Overall, these results seem to confirm the roles of the lipid, cholesterol, arachidonic acid, and drug metabolisms in the pathogeneses of atherosclerosis, CAD, and thrombosis, in cardiac damage, and ultimately in SCD, suggesting potential additional biomarkers of SCD, which would deserve further study.

### 4.2. Association with the Cause of Death (CoD)

When considering the three subgroups defined based on the autopsy findings (i.e., normal heart, CAD, and other known CoDs, as specified in Supplementary Table S4), some genetic variants were associated with a single category of SCD. The majority of the statistically significant associations were found for the subgroup of “other known CoD”, and the implied genes were involved in functions such as the cholesterol and drug metabolisms, but also cellular stress response, apoptosis, inflammation, immune response, and neurodevelopment or degeneration. The wide variability of the involved genes and functions was expected, given the fact that this subgroup of SCD is the most uneven in its composition: specific variants might be pathogenetic of specific cardiac structural modifications or diseases leading to SCD. On the other hand, the cholesterol and xenobiotic metabolisms were associated with both the “other known CoD” and CAD subgroups, suggesting that there might be common pathways involved in different kinds of SCD. Interestingly, cases of SCD with normal heart in the autopsy only demonstrated an association with possible variants when compared to the control cohort and not when looking at the intra-group comparison, but this might be due to the limited sample size. One of the two variants associated with the “normal heart” subgroup is located in the *EXOC6* gene and had not formerly been related to other phenotypes. *EXOC6*, encoding the exocyst complex component 6, is involved in translocation of the GLUT4 glucose transporter in adipocytes [74] and insulin secretion in pancreatic β-cells, increasing the risk of type 2 diabetes [75]. It would be interesting to further study the role of this gene and of glucose metabolism in SCD, even if there is currently no evidence of an association between EXOC6 and cardiovascular diseases such as atherosclerosis or CAD. It is necessary to emphasize that our SCD cohort widely differed from that of another study [76], where sudden arrhythmic death syndrome was identified as the cause of death in a majority of SCD cases. In contrast to Papadakis and colleagues [76], we detected no association between the “normal heart” subgroup and genetic variants located in the genes related to cardiac arrhythmias, such as *RYR2*, *CACNA1C,* and *SCN5A*. However, it is possible that the lack of association with arrhythmogenic genes might be due to the low sample number in our “normal heart” subgroup.

Further in-depth analyses are needed to elucidate the relationship between the genes involved in brain functions and SCD, since the autonomic nervous system contributes to the maintenance of the cardiovascular system’s homeostasis [77] and an imbalance in autonomic neural activity and remodeling enhances the risk of pathologies such as arrhythmias and heart failure up to sudden cardiac death [78]. Alzheimer’s disease is a neurodegenerative disorder also characterized by intraneural tangles of the tau protein encoded by *MAPT* [79,80], where the CAD subgroup of cases displayed a significance in a variant localized herein (rs17651507, Table 3). Notably, Alzheimer’s disease displayed a correlation with CAD and other cardiac dysfunctions [81], corroborating a likely involvement of this gene and the nervous system in SCD by promoting neural dysfunction. Further support for the implication of the nervous system in SCD was the statistical significance that we found in a variant (rs988748) in the *BDNF* gene through the case–control comparison; indeed, the neurotrophic factor encoded by this gene is highly related to cardiovascular disease development [82]. In addition, the rs17651507 variant in *MAPT* has been associated with waist–hip ratio [83], which could be in agreement with data showing a connection between cognitive impairment and obesity [84].

The present study confirmed and strengthened the roles of several genetic variants related to CAD, thrombosis, and risk factors for atherosclerosis in the determinism of SCD. Additional polymorphisms have been pinpointed as being related to SCD, mainly mapping in genes involved in the pathways of the lipid, cholesterol, arachidonic acid, and xenobiotics/drugs metabolisms. Considering the large number of variants and genes related to SCD reported herein, SCD appears as a rather polygenic trait, in which the normal and altered activities of many genes contribute to the pathogenesis of cardiovascular conditions leading to death. More in-depth and wide studies in forensic cases are required to clarify the significance of the biomarkers suggested herein, involved in cardiovascular functions, but not yet associated with cardiovascular diseases, in order to investigate their role in the pathogenesis of SCD and their potential roles in diagnostic tests. Given the fact that relatives and families of individuals who have died of SCD can be diagnosed with heritable conditions, such as Brugada syndrome [76], these results might also improve individual risk assessments, as well as screening and prevention for family members.

## 5. Conclusions

Thanks to the application of a genome-wide scan of sudden cardiac death, many variants that statistically differed with respect to the control cohort, which are likely implicated in SCD, were pinpointed. Some of these polymorphisms had already been detected as risk factors for the development of atherosclerosis, thrombosis, and coronary artery disease, thus strengthening their association with SCD. Even if most of these variants had not previously been associated with any character, many were found to be mapped in the genes involved in cardiovascular functions and pathologies. Furthermore, several biological pathways linked to the same diseases are enriched by these genes, pointing out that the lipid, cholesterol, arachidonic acid, and drug metabolisms are highly implied in SCD.

The three subgroups of SCD, as determined by autopsy examinations, were significantly differentiated only in a few genotypes. Despite the small sample number, these results provide the opportunity for further analyses relating to different causes of death.

Finally, owing to the large number of variants and genes related to SCD that we discovered in this study, our study supports that sudden cardiac death is a polygenic trait, in which the normal and altered activities of many genes contribute to the pathogenesis of cardiovascular conditions leading to death. Nevertheless, the current lack of involvement of many variants in cardiovascular diseases makes it necessary to investigate these polymorphisms further and more deeply, with the aim of clearly defining their roles in the pathogenesis of SCD and whether they will be useful as potential diagnostic markers allowing for prevention measures.

## Figures and Tables

**Table 1:** Descriptive statistic of the population.

Description		Normal Heart (n = 12)	Coronary Artery Disease (n = 10)	Other Known CoD (n = 8)	Total (n = 30)	*p*-Value
*Ancestry*	European	10	10	6	26	0.455
	Other	2	0	2	4	
*Age (mean, SD)*		38.5 (19.96)	53.5 (14.67)	38.3 (22.8)	43.4 (19.9)	0.147
*Gender*	M	10	10	7	27	0.415
	F	2	0	1	3	
*Activity before death*	Rest	9	7	3	19	0.222
	Moderate activity	2	3	2	7	
	Physical activity	-	-	2	2	
	Psychic stress	1	-	1	2	
*Medical history*	Neurologic/psychiatry	3	-	1	4	0.298
	RF	-	3	1	4	
	Substance use disorder	2	2	1	5	
	None	6	2	4	12	
	Unknown	1	3	1	5	
*Toxicology*	Positive	4	2	2	8	0.744
	Negative	8	8	6	22	

**Table 3:** Genetic variants showing statistically significant frequency difference in each autoptic subgroup.

Autoptic Subgroup	Variant	Type Variant	Gene	Gene Function	*p* ^1^	*p* ^2^
Other known CoD	rs10752613	Intergenic variant	-	-	0.022	0.014
	rs12986742	Intron variant	*LINC01122*	Long non-coding RNA	-	0.046
	rs6746883	Ncte variant *	*SULT1C2*	Drug metabolism	0.04	0.009
	rs16831114	Intergenic variant	-	-	0.04	-
	rs2602877	Intron variant	*LOC100507053*	Long non-coding RNA, alcohol addiction	0.031	0.001
	rs7734083	Intron variant	*RGS7BP*	Brain functions, neuropsychiatric disorders, drug addiction	0.019	0.041
	rs2092585	Ncte variant *	*LOC105374869*	Long non-coding RNA	-	0.027
	rs7741026	TF binding site	*CARMIL1* *SCGN*	Cellular components organization Cellular stress response, diabetes	-	0.03
	rs3131931	Intergenic variant	-	-	0.022	0.001
	rs831510	Missense variant	*FGD2*	Intracellular signaling	0.31	0.008
	rs747199	Intron variant	*SLC29A1* *POLR1C*	Adenosine transport across membranes RNA polymerase I and III subunit C	-	0.033
	rs7761731	Missense variant	*CYP39A1*	Cholesterol clearance, drug metabolism	0.016	0.013
	rs952884	Intron variant	*CYP39A1*	-	0.031	-
	rs9446917	Intron variant	*LOC124901342*	Long non-coding RNA	0.022	0.014
	rs12056033	Intergenic variant	-	-	0.016	0.018
	rs10091356	Intron variant	*LOC101929028*	Long non-coding RNA	-	0.039
	rs10087388	3′ UTR variant	*RNF170*	IP3 receptors degradation	-	0.042
	rs3747532	Missense variant	*CER1*	Embryonal development	-	0.042
	rs1818809	Intergenic variant	-	-	0.024	-
	rs1801041	3′ UTR variant	*DNA2*	DNA replication	-	0.026
	rs10500633	Intron variant	*MMP26*	Extracellular proteins cleavage, inflammation	0.04	0.013
	rs2024301	Missense variant	*CLEC4A*	Immune response	-	0.046
	rs10842971	Missense variant	*PZP*	Proteinase inhibition	0.016	0.008
	rs2306894	Missense variant	*CLEC1A*	Immune response	-	0.048
	rs1971911	3′ UTR variant	*DNM1L*	Mitochondrial and peroxisomal division, apoptosis	-	0.02
	rs2288035	3′ UTR variant	*WWOX*	Neurodegeneration, cholesterol and glucose metabolisms	-	0.044
	rs2228100	Missense variant	*ALDH3A1*	Xenobiotics metabolism, cornea protection	-	0.043
	rs12951993	Intergenic variant	-	-	0.02	0.024
	rs10409101	TF binding site	-	-	-	0.043
	rs12151363	Missense variant	*TDRD12*	piRNAs metabolic processes	0.046	-
	rs400058	Intron variant	*CADM4*	Cell–cell adhesion	0.04	-
	rs4148125	Intron variant	*ABCG1*	Cholesterol metabolism	-	0.007
Coronary artery disease	rs6746883	Ncte variant *	*SULT1C2*	Drug metabolism	-	0.036
	rs4685744	3′ UTR variant	*SUMF1*	Protein metabolism	0.029	0.018
	rs2477642	Intron variant	*MRC1*	Glycoprotein endocytosis by macrophages	-	0.042
	rs10852287	Intergenic variant	-	-	0.031	0.018
	rs17651507	Intron variant	*MAPT*	Tau protein, neurodegenerative disorders	0.013	0.009
Normal heart	rs1551634	Intergenic variant	-	-	-	0.046
	rs4933754	Intron variant	*EXOC6*	Exocytosis, glucose metabolism	-	0.04

^1^ *p*-value of Fisher test performed in intra-autoptic groups comparison. ^2^ *p*-value of Fisher test performed in subgroups–controls comparison. * Ncte variant = Non-coding transcript exon variant.

## Data Availability

Data sharing not applicable.

## Supplementary Materials

The following supporting information can be downloaded at: https://www.mdpi.com/article/10.3390/genes14061265/s1, Table S1: Table containing results of chi-square performed in case–control comparison. All reported variants have *p*-value ≤ 0.001; Table S2: Table containing all genes already associated with some degree with cardiovascular functions and diseases; Table S3: Table containing results of pathway enrichment analysis, displaying only pathways with *p*-adjusted ≤ 0.05. Table S4: Breakdown of the other “highly probable” causes of death identified.
